# Prognostic consequences of implementing cancer patient pathways in Denmark: a comparative cohort study of symptomatic cancer patients in primary care

**DOI:** 10.1186/s12885-017-3623-8

**Published:** 2017-09-06

**Authors:** Henry Jensen, Marie Louise Tørring, Peter Vedsted

**Affiliations:** 1 Research Centre for Cancer Diagnosis in Primary Care, Research Unit for General Practice, Department of Public Health, Aarhus University, Bartholins Allé 2, DK-8000 Aarhus C, Denmark; 2 Department of Anthropology, School of Culture and Society, Aarhus University, Moesgaard Allé 20, DK-8270 Hoejbjerg, Denmark

**Keywords:** Urgent referral, Neoplasm, (early) diagnosis, General practice, Survival, Mortality, Denmark

## Abstract

**Background:**

Cancer Patient Pathways (CPPs) were introduced in 2000–2015 in several European countries, including Denmark, to reduce the time to diagnosis and treatment initiation and ultimately improve patient survival. Yet, the prognostic consequences of implementing CPPs remain unknown for symptomatic cancer patients diagnosed through primary care.

We aimed to compare survival and mortality among symptomatic patients diagnosed through a primary care route before, during and after the CPP implementation in Denmark.

**Methods:**

Based on data from the Danish Cancer in Primary Care (CaP) Cohort, we compared one- and three-year standardised relative survival (RS) and excess hazard ratios (EHRs) before, during and after CPP implementation for seven types of cancer and all combined (*n* = 7725) by using life-table estimation and Poisson regression. RS estimates were standardised according to the International Cancer Survival Standard (ICSS) weights. In addition, we compared RS and EHRs for CPP and non-CPP referred patients to consider potential issues of confounding by indication.

**Results:**

In total, 7725 cases were analysed: 1202 before, 4187 during and 2336 after CPP implementation. For all cancers combined, the RS_3years_ rose from 45% (95% confidence interval (CI): 42;47) before to 54% (95% CI: 52;56) after CPP implementation. The excess mortality was higher before than after CPP implementation (EHR_3years_ before vs. after CPP = 1.35 (95% CI: 1.21;1.51)). When comparing CPP against non-CPP referred patients, we found no statistically significant differences in RS, but we found lower excess mortality among the CPP referred (EHR_1year_ CPP vs. non-CPP = 0.86 (95% CI: 0.73;1.01)).

**Conclusion:**

We found higher relative survival and lower mortality among symptomatic cancer patients diagnosed through primary care after the implementation of CPPs in Denmark. The observed changes in cancer prognosis could be the intended consequences of finding and treating cancer at an early stage, but they may also reflect lead-time bias and selection bias. The finding of a lower excess mortality among CPP referred compared to non-CPP referred patients indicates that CPPs may have improved the cancer prognosis independently.

## Background

Cancer survival varies between countries [1–4]. It appears to be lower in countries where general practitioners (GPs) are assigned the role as first point of contact to the health services and gatekeeper to specialised cancer care [3, 5, 6]. Delayed referrals from primary care and/or delayed cancer diagnoses may explain some of the variation in survival between countries. Therefore, many countries with gatekeeper systems have sought to increase the survival by implementing comprehensive national cancer guidelines, such as the English *NICE Guidance*, the Scottish *SIGN Guidelines* and the Danish *Cancer Patient Pathways* (CPPs) [7–15].

The prognostic benefits from implementing CPPs remain unknown for symptomatic cancer patients diagnosed through primary care, although this group constitutes more than 75% of all cancer patients [16, 17]. The few existing studies are too small and underpowered to detect changes in survival [18–20], or they fail to recognise important issues of selection and confounding by indication related to the radical changes in referral routes [21–26].

Another methodological concern regards lead-time bias and the use of survival as an effect measure. Previous findings of increased survival after CPP implementation could be a sign that CPPs have advanced the date of diagnosis to an earlier point in time without postponing the patient’s time of death [27]. Problems of lead time bias may be mitigated by calculating the mortality instead of the survival, but no studies of CPP implementation have done this so far.

The aim of this study was firstly to compare survival and mortality among symptomatic patients diagnosed through a primary care route across the time (i.e. before, during and after) of CPP implementation in Denmark – for seven common cancer types. Secondly, we aimed to compare CPP and non-CPP referred patients in terms of survival and mortality to acknowledge and determine issues of confounding by indication.

## Methods

Data from GPs and registries recorded in the Danish Cancer in Primary Care (CaP) cohort [28] were used to compare survival and mortality between three cohorts of incident cancer patients diagnosed through a primary care route before, during and after CPP implementation.

### Setting

The study took place in Denmark, where the publicly funded health-care system ensures free access to diagnostic procedures and treatment for all citizens. Almost all citizens (>98%) are registered with a GP, who acts as a gatekeeper to the rest of the health-care system (except for emergencies and private practice otorhinolaryngologists and ophthalmologists who can be accessed directly) [29].

The Danish CPP guidelines list specific criteria for urgent referral and describe well-defined diagnostic entities until treatment, including limited time frames [8]. The Danish CPPs were introduced by national law in October 2007 and sequentially implemented throughout 2008 and 2009; by April 2008 CPPs for breast, colorectal, lung and head and neck cancers were implemented, by June 2008 CPPs for gynaecological cancers were implemented, by September 2008 CPPs for leukemic cancers were implemented, by November 2008 CPPs for urinary tract, malignant melanoma, brain and CNS cancers were implemented, and by January 2009 CPPs for prostate, upper gastrointestinal, and remaining cancers were implemented [30].

Breast cancer patients were deemed ineligible for inclusion in the present study because a national screening programme for this type of cancer was implemented in Denmark in 2007–2009. Likewise, we excluded prostate cancer patients due to increased use of prostate specific antigen (PSA) tests in general practice throughout the study period [31], which increased the proportion of prostate cancer patients with localised tumours, but these were unrelated to the CPP implementation [32, 33].

### Patient population and data collection

Identification of patients, data collection and drop-out analysis have been described in detail elsewhere [28, 34]. In brief, patients were identified in hospital registers and in the Danish National Patient Registry before (1 September 2004–31 August 2005), during (1 October 2007–30 September 2008) and after (1 May – 31 August 2010) CPP implementation.

Patients were eligible if they were 18 years of age or older, were listed with a GP, attended general practice as part of their diagnostic route and were registered with a verified first-time diagnosis of colorectal cancer (ICD-10: C18-C20), lung cancer (ICD-10: C34), malignant melanoma (ICD-10: C43), head and neck cancer (ICD-10: C01–14, C30-C32, C462 & C73), upper gastrointestinal (upper GI) cancer (ICD-10: C15-C17 and C22-C26), gynaecological cancer (ICD-10: C51-C58) or urinary system cancer (ICD-10: C64-C68).

**Fig. 1:**
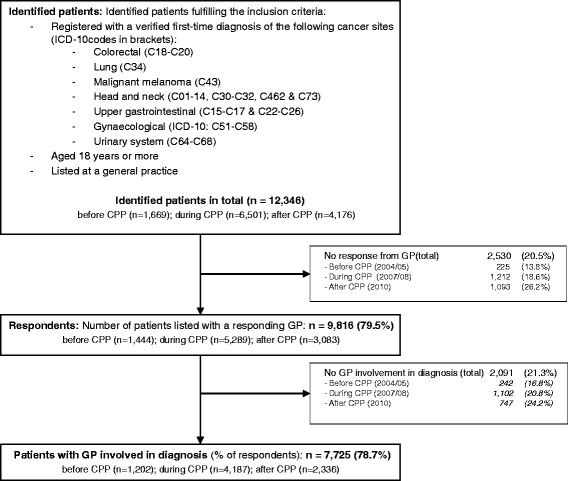
Flow of patients in study

A questionnaire was sent to each patient’s GP. The GP was asked to provide a detailed description of the patient’s diagnostic pathway on the basis of the electronic medical record and discharge letters from hospitals and specialists. This information allowed us to group patients diagnosed after CPP implementation into ‘CPP-referred patients’ and ‘non-CPP referred patients’ [28]. The GPs responded for 9816 (80%) of the 12,346 identified incident cancer patients [34] (Fig. 1). Patients with responding GPs were less likely to be males and had fewer missing data on tumour stage than the other patients (data not shown) [34]. Responding GPs reported on the basis of the question: “Were you/your general practice involved in diagnosing the cancer?” to be involved in diagnosing cancer for 7725 (79%) of the cases [28, 34] (Fig. 1). Subsequently, the study population was restricted to the 79% of patients who had attended general practice before the cancer diagnosis.

### Defining outcome, exposure and covariates

The study outcome was death. From the Danish Civil Registration System, we retrieved information on migration and death. All patients were followed for at least three years after diagnosis. When we compared survival (rates) and (excess) mortality in patients before, during and after CPP implementation, the date of diagnosis was obtained from the Danish Cancer Registry and corresponds to the first contact to a hospital (i.e. admission date). If the patient was diagnosed by a private practicing specialist, the date of diagnosis corresponds to the date of the clinical diagnosis [35].

The exposure of the study was CPP implementation status defined according to the sampling time for the three sub-cohorts: 2004/05 = before, 2007/08 = during and 2010 = after CPP implementation. The after CPP cohort was subdivided into ‘CPP referred’ and ‘non-CPP referred’ patients based on GP-reported information on referral route [28].

The co-variates used in the analyses were, sex, age, comorbidity, tumour stage, educational level and disposable income. Sex and age was derived from the Danish civil registration (CPR) number [36]. Comorbidity was calculated by information from the Danish National Patient Registry ten years prior to cancer diagnosis using the Charlson Comorbidity Index (excluding the cancer in question) and categorised into none, low (score 1–2) and high (score ≥ 3) [28]. Tumour stage for colorectal, lung, malignant melanoma and bladder cancers was categorised using established cancer-specific algorithms to classify tumours with missing TNM components in the Danish Cancer Registry as either: local, regional, distant, unknown or missing [37–40]. TNM staging information for the remaining patients was categorized using the following principle: local (no positive lymph nodes or metastasis), regional (positive lymph nodes), distant (metastatic cancer), missing (no T, N, and M information) and unknown for the remaining cancers [28]. Information on educational level was obtained from Statistics Denmark and grouped according to the International Standard Classification of Education (ISCED) [26] into ‘low’ (ISCED levels 1 and 2), ‘medium’ (ISCED levels 3 and 4) and ‘high’ (ISCED levels 5 and 6). Likewise, information on OECD household disposable income in the year prior to the diagnosis was obtained from Statistics Denmark and grouped into tertiles: ‘low’, ‘medium’ and ‘high’.

### Statistical analysis

We analysed the one- and three-year relative survival rates and the excess mortality for each of the seven cancer types and for all combined.

Relative survival (RS) was computed by life-table estimation (i.e. complete approach) and expressed as percentages. We used the Ederer II method to determine the expected survival [37]. The lifetables used to account for the underlying mortality were sex-, age- and year-specific and these are freely accessible from the home page of Statistics Denmark [41]. The survival estimates were calculated at monthly intervals up to three years. Estimates of the relative survival were standardised using the International Cancer Survival Standard (ICSS) weights [42].

To determine the association between cohort time (i.e. CPP implementation status) and prognosis, while accounting for possible confounders, Excess Hazard Ratios (EHRs) were computed using a generalised linear model with Poisson linkage. Univariable and multivariable models were built for each cancer type and for all cancers combined. Multivariable models controlled for the effects of sex, age, cancer type (models for all cancers combined only), tumour stage, comorbidity, educational level and disposable income. Additionally, for gynaecological cancers, we also took into account whether the cancer was an ovarian cancer or not.

A statistical level of *p* ≤ 0.05 was considered significant in all analyses. Assessment of statistically significant differences in the relative survival between groups were done by comparing confidence limits (if the confidence intervals did not overlap, a statistically significant difference existed). Analyses were done using Stata® statistical software, version 14 (StataCorp LP, College Station, TX, USA).

## Results

Of the 7725 study subjects, 1202 were diagnosed before, 4187 during and 2336 after the CPP implementation (Fig. 1, Table 1). The after-CPP cohort consisted of 772 (33%) CPP referred and 1564 (67%) non-CPP referred patients. Patient characteristics are displayed in Table 1.

**Table 1:** Patient characteristics by CPP implementation status and referral status

	Before CPP		During CPP		After CPP					
					Total		Non-CPP referred		CPP referred	
	n	(%)	n	(%)	n	(%)	n	(%)	n	(%)
Total	1202	(100)	4187	(100)	2336	(100)	1564	(100)	772	(100)
Deaths in three years	695	(57.8)	2208	(52.7)	1165	(49.9)	775	(49.6)	390	(50.5)
Survival rate (raw)										
1 year	0.592		0.647		0.671		0.669		0.675	
3 years	0.413		0.471		0.501		0.505		0.495	
Sex										
Woman	624	(51.9)	2120	(50.6)	1128	(48.3)	782	(50.0)	346	(44.8)
Man	578	(48.1)	2067	(49.4)	1208	(51.7)	782	(50.0)	426	(55.2)
Age, median (IQI)	68	(58–77)	68	(59–76)	68	(59–76)	68	(59–76)	68	(60–76)
Age groups (years)										
18–44	84	(7.0)	258	(6.2)	141	(6.0)	102	(6.5)	39	(5.1)
45–54	138	(11.5)	469	(11.2)	264	(11.3)	191	(12.2)	73	(9.5)
55–64	293	(24.4)	1040	(24.8)	549	(23.5)	353	(22.6)	196	(25.4)
65–74	337	(28.0)	1235	(29.5)	724	(31.0)	472	(30.2)	252	(32.6)
75-	350	(29.1)	1185	(28.3)	658	(28.2)	446	(28.5)	212	(27.5)
Diagnoses										
CRC	283	(23.5)	1073	(25.6)	629	(26.9)	405	(25.9)	224	(29.0)
Lung	280	(23.3)	1018	(24.3)	501	(21.4)	299	(19.1)	202	(26.2)
Melanoma	125	(10.4)	403	(9.6)	236	(10.1)	154	(9.8)	82	(10.6)
Head & neck	74	(6.2)	260	(6.2)	180	(7.7)	141	(9.0)	39	(5.1)
Upper GI	185	(15.4)	570	(13.6)	336	(14.4)	252	(16.1)	84	(10.9)
Gynaecological	141	(11.7)	484	(11.6)	250	(10.7)	186	(11.9)	64	(8.3)
Urinary system	114	(9.5)	379	(9.1)	204	(8.7)	127	(8.1)	77	(10.0)
Tumour stage										
Local	452	(37.6)	1530	(36.5)	868	(37.2)	596	(38.1)	272	(35.2)
Regional	219	(18.2)	807	(19.3)	458	(19.6)	289	(18.5)	169	(21.9)
Distant	330	(27.5)	1294	(30.9)	719	(30.8)	462	(29.5)	257	(33.3)
Unknown/missing	201	(16.7)	556	(13.3)	291	(12.5)	217	(13.9)	74	(9.6)
Comorbidity										
None	793	(66.0)	2913	(69.6)	1.636	(70.0)	1.064	(68.0)	572	(74.1)
Moderate	319	(26.5)	1051	(25.1)	563	(24.1)	394	(25.2)	169	(21.9)
High	90	(7.5)	223	(5.3)	137	(5.9)	106	(6.8)	31	(4.0)
Educational level										
Low	473	(39.4)	1874	(44.8)	897	(38.4)	587	(37.5)	310	(40.2)
Medium	421	(35.0)	1450	(34.6)	883	(37.8)	601	(38.4)	282	(36.5)
High	202	(16.8)	641	(15.3)	456	(19.5)	307	(19.6)	149	(19.3)
Missing	106	(8.8)	222	(5.3)	100	(4.3)	69	(4.4)	31	(4.0)
Household income										
Low	378	(31.4)	1323	(31.6)	778	(33.3)	505	(32.3)	273	(35.4)
Medium	363	(30.2)	1364	(32.6)	802	(34.3)	542	(34.7)	260	(33.7)
High	395	(32.9)	1360	(32.5)	753	(32.2)	513	(32.8)	239	(31.0)

### Survival and excess mortality across the time of CPP implementation

Patients diagnosed after CPP implementation had higher one- and three-year relative survival (RS_1year_ and RS_3year_) than patients diagnosed before CPP implementation for each of the seven types of cancer, with statistically significant differences for lung cancer, gynaecological cancers and all cancers combined (Tables 2 and 3).

**Table 2:** One-year relative survival (RS) expressed as percentages with 95% confidence interval (95%CI)

	Before CPP		During CPP		After CPP					
					Total		Non-CPP referred		CPP referred	
	RS	(95%CI)	RS	(95%CI)	RS	(95%CI)	RS	(95%CI)	RS	(95%CI)
CRC	79.5	(74.0;84.0)	80.9	(78.1;83.4)	82.0	(78.4;85.0)	82.9	(78.4;86.5)	80.1	(74.0;85.7)
Lung	31.7	(26.6;36.9)	38.7	(35.4;42.0)	43.7	(39.6;47.8)	40.5	(35.4;45.6)	48.7	(41.7;55.3)
Melanoma	93.9	(87.7;97.0)	96.9	(93.9;98.5)	96.5	(92.2;98.4)	97.9	(90.9;99.5)	92.9	(83.7;97.0)
Head & neck	77.1	(63.5;87.1)	87.3	(80.5;91.8)	83.1	(75.2;88.7)	86.8	(78.0;92.2)	70.0	(52.5;82.1)
Upper GI	31.3	(24.9;38.0)	38.5	(34.6;42.3)	38.6	(33.4;43.8)	37.6	(31.6;43.5)	^a^	
Gynaecological	76.9	(68.5;83.3)	84.7	(80.7;87.9)	90.7	(85.5;94.1)	89.3	(82.7;93.5)	95.3	(84.3;98.7)
Urinary system	65.8	(56.4;73.6)	74.1	(69.1;78.4)	77.3	(69.7;83.2)	73.7	(64.1;81.2)	^a^	
Total	60.7	(57.8;63.4)	66.5	(65.0;68.0)	69.0	(67.1;70.9)	68.7	(66.3;70.9)	69.8	(66.4;73.0)

RS estimates are calculated using the complete approach and standardised using ICSS weights. Underlying mortality was accounted for using life tables. ^a^Could not be computed due to a low number of cases

**Table 3:** Three-year relative survival (RS) expressed as percentages with 95% confidence interval (95%CI)

	Before CPP		During CPP		After CPP					
					Total		Non-CPP referred		CPP referred	
	RS	(95%CI)	RS	(95%CI)	RS	(95%CI)	RS	(95%CI)	RS	(95%CI)
CRC	63.8	(57.0;69.9)	66.4	(62.9;69.7)	69.3	(64.8;73.3)	70.8	(65.2;75.7)	65.4	(57.6;72.1)
Lung	11.3	(8.00;15.4)	16.2	(13.7;18.9)	20.4	(15.6;25.7)	19.5	(13.6;26.2)	20.9	(15.5;26.9)
Melanoma	89.6	(81.5;94.3)	91.7	(87.4;94.5)	91.9	(86.1;95.4)	95.6	(87.0;98.5)	85.3	(74.2;91.8)
Head & neck	57.0	(41.5;69.8)	70.3	(61.6;77.4)	73.6	(64.1;81.0)	77.8	(66.8;85.6)	58.5	(39.2;73.6)
Upper GI	18.5	(13.5;24.2)	19.8	(16.5;23.3)	18.5	(14.5;22.9)	17.4	(12.9;22.5)	^a^	
Gynaecological	58.3	(48.7;66.8)	70.7	(67.1;77.4)	75.2	(68.2;80.8)	72.8	(64.5;79.5)	84.4	(70.8;92.0)
Urinary system	47.7	(38.5;56.3)	59.9	(54.2;65.1)	61.7	(53.1;69.3)	59.2	(48.6;68.4)	^a^	
Total	44.5	(41.5;47.5)	51.0	(49.4;52.6)	54.4	(52.2;56.5)	54.5	(51.8;57.1)	54.1	(50.3;57.8)

RS estimates are calculated using the complete approach and standardised using ICSS weights. Underlying mortality was accounted for using life tables. ^a^Could not be computed due to a low number of cases

The excess mortality ratios at one- and three-year follow-up (EHR_1year_ & EHR_3year_) were higher before than after CPP implementation for all cancer types (EHR_1year_ = 1.25 (95% CI: 1.10;1.43) & EHR_3years_ = 1.35 (95% CI: 1.21;1.51)), with statistically significant differences for lung cancer, gynaecological cancers and all cancers combined (Tables 4 and 5).

**Table 4:** One-year Excess Hazard Ratios (EHR) and 95% confidence intervals (95%CI) according to implementation of standardised cancer patient pathways (CPP) in Denmark

	Before CPP		During CPP		After CPP			
					Total		CPP referred	
	EHR	(95%CI)	EHR	(95%CI)	EHR	(95%CI)	EHR	(95%CI)
CRC	1.02	(0.69;1.51)	1.04	(0.80;1.34)	1	ref	1.15	(0.76;1.75)
Lung	1.11	(0.90;1.37)	1.01	(0.87;1.17)	1	ref	**0.73**	(0.57;0.94)
Melanoma	1.13	(0.21;5.79)	0.84	(0.24;2.94)	1	ref	0.62	(0.09;4.35)
Head & neck	1.74	(0.82;3.67)	1.03	(0.55;1.94)	1	ref	1.22	(0.44;3.33)
Upper GI	1.24	(0.97;1.59)	0.94	(0.78;1.13)	1	ref	0.96	(0.68;1.34)
Gynaecological	**2.60**	(1.37;4.94)	1.29	(0.75;2.22)	1	ref	0.47	(0.11;1.97)
Urinary system	1.59	(0.96;2.66)	0.95	(0.64;1.41)	1	ref	0.51	(0.25;1.06)
Total	**1.25**	(1.10;1.43)	0.99	(0.90;1.10)	1	ref	0.86	(0.73;1.01)

Last column shows EHRs and 95%CIs between referral route (CPP or not) in 2010

EHRs adjusted for sex, age, tumour stage, comorbidity (Charlson’s Comorbidity Index), educational level, disposable income, diagnosis (total only) and ovarian cancer (gynaecological cancers only). Estimates in bold indicate a statistical significance of *p* < 0.05 or less

**Table 5:** Three-year Excess Hazard Ratios (EHR) and 95% confidence intervals (95%CI) according to implementation of standardised cancer patient pathways (CPP) in Denmark

	Before CPP		During CPP		After CPP			
					Total		CPP referred	
	EHR	(95%CI)	EHR	(95%CI)	EHR	(95%CI)	EHR	(95%CI)
CRC	1.16	(0.87;1.57)	1.11	(0.91;1.36)	1	ref	1.13	(0.81;1.57)
Lung	**1.30**	(1.09;1.55)	1.13	(0.99;1.28)	1	ref	**0.77**	(0.62;0.95)
Melanoma	1.11	(0.48;2.55)	0.64	(0.31;1.32)	1	ref	1.97	(0.65;5.97)
Head & Neck	2.12	(1.26;3.56)	1.25	(0.79;1.97)	1	ref	1.36	(0.62;2.98)
Upper GI	1.15	(0.92;1.43)	0.93	(0.79;1.10)	1	ref	1.00	(0.74;1.34)
Gynaecological	**1.99**	(1.29;3.07)	1.15	(0.81;1.65)	1	ref	0.81	(0.40;1.62)
Urinary system	1.49	(0.98;2.26)	0.93	(0.68;1.27)	1	ref	0.67	(0.40;1.12)
Total	**1.35**	(1.21;1.51)	1.06	(0.98;1.15)	1	ref	0.91	(0.79;1.04)

Last column shows EHRs and 95%CIs between referral route (CPP or not) in 2010

EHRs adjusted for sex, age, tumour stage, comorbidity (Charlson’s Comorbidity Index), educational level, disposable income, diagnosis (total only) and ovarian cancer (gynaecological cancers only). Estimates in bold indicate a statistical significance of *p* < 0.05 or less

### Survival and excess mortality between referral routes

For all cancers combined, we saw no statistically significant differences in RS_1year_ or RS_3year_ between CPP-referred and non-CPP referred patients (Tables 2 and 3). However when looking at the individual cancer types we found a better survival for CPP-referred than for non-CPP referred patients among lung and gynaecological cancers (Tables 2 and 3).

When we compared the excess mortality between CPP and non-CPP referred patients, an overall trend of lower excess mortality was observed among CPP-referred patients compared to non-CPP referred patients (EHR_3years_ = 0.91 (95% CI: 0.79;1.04)) (Tables 4 and 5), with statistically significantly lower excess mortality only among lung cancer patients (EHR_3years_ = 0.77 (95% CI: 0.62;0.65)) (Tables 4 and 5). Although the EHRs for all cancers combined were lower for CPP referred patients, two cancer types (colorectal and head/neck) displayed an EHR_1year_ higher than one (Table 4), and only three cancer types (lung, gynaecological, and urinary system) displayed an EHR_3year_ of less than one (Table 5).

## Discussion

We found improved prognosis for symptomatic cancer patients diagnosed through a primary care route after CPP implementation in Denmark for seven different cancer types, both in terms of higher relative survival and lower excess mortality. The findings were only statistically significant overall and for lung and gynaecological cancers separately. CPP referred patients did not have statistically significantly higher survival than non-CPP referred patients, but CPP referred patients tended to have a lower excess mortality for all cancers combined.

### Strengths and limitations

The study’s strengths include a large sample size, the population-based design permitted by the uniformly organised healthcare system in Denmark and the complete follow-up through population-based registries, which limited the risks of selection and information bias. The high response rate among GPs (79%) also reduced the potential for selection bias. By excluding patients for whom the GP had not been involved in the diagnosis, we ensured a more homogeneous group to evaluate the possible effect of CPP implementation on the target population of symptomatic cancer patients presenting in primary care; we thus obtained better internal validity. Furthermore, the analyses were strengthened by addressing lead-time bias and confounding by indication as discussed further below.

This study also has limitations. Firstly, 21% of the study base could not be included in the final analyses because of GP non-response. We have no reason to believe that GPs became more or less inclined to participate over time due to the patient’s survival status. All three cohorts were found to be representative of incident cancer patients in Denmark at the time of inclusion [28]. This indicates that any selection bias is likely to be non-differential, and our estimates may thus underestimate the real association.

Secondly, lead time bias may be at play because a more timely diagnosis (due to CPP implementation) have advanced what would have been the original date of diagnosis to an earlier point in time [11, 43, 44], but this may not necessarily have delayed the patient’s time of death [27]. This could have inflated the survival measures among CPP patients. Indeed, a recent study reports that lead time inferred from CPP implementation is at play in the cohorts used in this study [45]. Yet the lead time accounts for less than 15% of the increase in one-year survival rate, indicating that the survival rate did in fact improve across the time of CPP implementation in Denmark [45]. Together with our finding of corresponding lower excess hazard ratios, it suggests that the cancer prognosis did improve across time of the CPP implementation in Denmark.

Thirdly, studies of prognosis and use of CPPs may be prone to confounding by indication because CPP guidelines prioritize patients with specific signs and symptoms of cancer who are inherently more sick [18, 34, 46, 47]. We tried to disclose this problem by comparing prognosis between referral groups as the prioritization of more ill patients to the CPP route, should, hypothetically, incur that CPP referred patients have lower relative survival and higher excess mortality than non-CPP referred patients.

Fourthly, residual confounding may have resulted from imperfect adjustment and potential misclassification of one or more confounding variables. Yet, the risk of residual confounding should be equally distributed for all cohorts in this study and lead to an underestimation of the true associations. We used benchmark registries and approaches to produce comparable stage information, but some misclassification may still have occurred due to missing information on staging as this data became more complete during the period of the CPP implementation [34, 37–40, 48–50]. We included missing stage as a separate category in the analyses to reduce this problem. Thus, the main effect of this misclassification would be increased variation and hence loss of statistical precision. The fact that we observed no major change in the estimates when controlling for measured comorbidity, income, educational level and tumour stage also speaks against the presence of residual confounding.

Finally, although cancer-specific analyses and the CPP/non-CPP stratification procedure were used to limit and acknowledge the risk of confounding and selection bias, the procedures also reduced the statistical precision of the study. A larger study is needed to assess the consistent, but not statistically significant cancer-specific effects found in this study.

### Comparison with other studies

Relative survival rates have increased since the mid-1990s in Denmark and many other countries [1–3, 51]. Still, the observed changes in the one-year relative survival among primary-care patients of more than eight percentage point, which we report in this study, are above the changes reported for all cancer patients (irrespective of diagnostic route) of approximately six percentage points from 2004 to 2010 in Denmark [2, 4]. Recent evidence suggest that only 15% (i.e. 0.8 percentage points) of the improvement in survival can be explained by lead time bias from the expedited diagnosis in the CPPs [45]. This indicates that something extraordinary in the handling of symptomatic cancer patients did take place within the Danish health-care system during the investigated period of time; the implementation of CPPs being the most tangible one.

The few previous studies on the prognostic effect of urgent referrals among symptomatic cancer patients diagnosed through primary care display diverging results [18–26, 34], which contrast our overall findings of improved prognosis across the time of the CPP implementation. A few of the previous studies did not observe a difference in prognosis [18–20, 34]. Some studies concluded that urgent referrals either improved or worsened the prognosis, but they did not take into account the important issues of lead time bias and confounding by indication [21–26]. Our findings of no statistically significant difference in the relative survival for colorectal cancer patients are in line with two studies from the UK on the impact of urgent referrals [18, 22]. These results contrast the findings from a small single-centre study from Denmark, which shows an improvement in the long-term absolute survival after compared to before CPP implementation [52]. The previously reported relative survival for all lung cancer patients in Denmark is slightly lower than that reported in our study [2]. This may be because lung cancer patients diagnosed through a primary care route (68%) are younger and have lower levels of comorbidity than lung cancer patients diagnosed through other routes [53]. Yet, our findings of lower excess mortality across the time of the CPP implementation correspond to recently published data from the Danish Lung Cancer Register [54]. The previously reported relative survival rates for malignant melanoma in Denmark [2] are similar to our findings across time, but no other study has so far investigated whether there is a difference in the relative survival between referral routes (whether CPP or not). Hence, we need further investigation of the interesting finding that the excess mortality among CPP referred patient with malignant melanoma was lower for the short term and higher for the long term when compared to non-CPP referred patients.

### Interpretation and underlying mechanisms

We know that the time to diagnosis and treatment decreased from before to after CPP implementation [11, 43, 44] and that these time intervals are shorter among patients with alarm symptoms of cancer [43, 55]. We also know that a range of other changes occurred in the health-care system during the study period (e.g. centralisation of cancer treatment) [8, 56], which may explain part of the findings. The centralisation of cancer treatment at fewer and more specialised hospitals in Denmark simultaneously with the CPP implementation may be a plausible reason for the improved prognosis [9, 51, 57–59]; greater centralisation of treatment infers higher volume of surgical procedures, which improves outcomes [60].

The findings that the time to diagnosis and treatment has decreased across the time of the CPP implementation [11, 43, 44] together with the improved survival fit well with the increasing evidence that time to diagnosis matters for the prognosis [61–64]. Furthermore, the concurrent decrease in excess mortality seen across the time of the CPP implementation in this study, together with the small effect of lead time on the improvement in survival [45], suggests that the CPP implementation has contributed to the improved prognosis, despite issues of lead time bias prevails in this study. Thus, it seems valid to assume that the CPP implementation has caused at least part of the higher relative survival and the lower excess mortality across time.

CPP referred patients due to being more ill at the time of referral [18, 34, 46, 47] were expected to have had lower relative survival than non-CPP referred patients due to confounding by indication. However, this was not supported by the finding that CPP referred and non-CPP referred patients displayed similar survival. Yet, this may be caused by lead time bias as patients referred to a CPP route have shorter time to diagnosis/treatment for cancer than non-CPP referred patients [11, 19, 43, 44, 52, 55]. This raises a principal problem; if the results are biased, we cannot trust a prognostic evaluation based solely on relative survival in a cross-sectional study design. However, as the results in our study are consistent with both an increase in the relative survival and a lower excess mortality across time, together with a trend towards lower excess mortality among CPP referred patients, it seems feasible that CPP implementation have, at least partially, improved the prognosis.

## Conclusion

This study supports the hypothesis that the prognosis of symptomatic cancer patients diagnosed through a primary care route has improved across the time of CPP implementation in Denmark, both in terms of higher survival and lower excess mortality. The observed changes in cancer prognosis could be the intended consequences of finding and treating cancer at an early stage, but they may also reflect lead-time bias and selection bias. The finding of lower excess mortality among CPP referred compared to non-CPP referred patients indicates that the CPPs improved the cancer prognosis independently. Yet, the improvement in the prognosis is also dependent on other factors than CPP guidelines, such as centralization of treatment.

## Abbreviations

CaP: the Danish Cancer in Primary Care Centre
CNS: Central Nervous System
CPP: Cancer Patient Pathway
CPR: Danish civil registration number
EHR: Excess Hazard Ratio
GP: General Practitioner
ICD: International Classification of Diseases
ICSS: International Cancer Survival Standard
ISCED: International Standard Classification of Education
NICE: the National Institute for Health and Care Excellence
OECD: The Organisation for Economic Co-operation and Development
RS: Relative survival
SIGN: Scottish Intercollegiate Guidelines Network
TNM: Tumour, node, Metastasis
UK: United Kingdom
